# Supplementary material for: The Planemo toolkit for developing, deploying, and executing scientific data analyses in Galaxy and beyond
Source: Genome Res. 2023 Feb;33(2):261–8. doi: 10.1101/gr.276963.122 (PMC10069471; doi:10.1101/gr.276963.122)
Supplement: Supplemental Material [file supp_33_2_261__DC1.html]

The Planemo toolkit for developing, deploying, and executing scientific data analyses in Galaxy and beyond — The Planemo toolkit for developing, deploying, and executing scientific data analyses in Galaxy and beyond — Supplemental Material 

# The Planemo toolkit for developing, deploying, and executing scientific data analyses in Galaxy and beyond

## Supplemental Material

- Supplemental\_Code.tar.gz
