# Supplementary material for: The Planemo toolkit for developing, deploying, and executing scientific data analyses in Galaxy and beyond
Source: Genome Res. 2023 Feb;33(2):261–8. doi: 10.1101/gr.276963.122 (PMC10069471; doi:10.1101/gr.276963.122)
Supplement: Supplemental Material [file supp_gr.276963.122_Supplemental_Code.tar.gz › planemo-0.75.3/docs/galaxy_changelog.html]

Galaxy Tool Framework Changes


class: center, middle
layout: true
class: inverse, middle
---
class: title
# Galaxy Tool Framework Changes
## John Chilton
This document describes changes to Galaxy's tooling framework over recent
releases.
---
### 16.04
Full [Galaxy changelog](https://docs.galaxyproject.org/en/master/releases/16.04\_announce.html).
---
#### Tool Profile Version ([PR #1688](https://github.com/galaxyproject/galaxy/pull/1688))
Tools may (and should) now declare a `profile` version (e.g.
`<tool profile="16.04" ...>`).
This allows Galaxy to fire a warning if a tool uses features too new for the
current version and allows us to migrate away from some undesirable default
behaviors that were required for backward compatiblity.
---
#### `set -e` by default ([d020522](https://github.com/galaxyproject/galaxy/pull/1688/commits/d020522650a9bfc86c22923a01fd5d7c07c65326))
From the [IUC best practices documentation](http://galaxy-iuc-standards.readthedocs.org/en/latest/best\_practices/tool\_xml.html#command-tag):
> \_"If you need to execute more than one shell command, concatenate them with a double ampersand (`&&`), so that an error in a command will abort the execution of the following ones."\_
The job script generated with profile `16.04`+ tools will include a `#set -e` statement causing this behavior by default.
Older-style tools can enable this behavior by setting `strict="true"` on
the tool `command` XML block.
---
#### Using Exit Codes for Error Detection ([b92074e](https://github.com/galaxyproject/galaxy/commit/b92074e6ff87a19133b4d973577779c4ee6286d7))
Previously the default behavior was for Galaxy to ignore exit codes and
declare a tool in error if issued any output on standard error. This was
a regretable default behavior and so all tools were encouraged to declare
special XML blocks to force the use of exit codes.
For any tool that declares a profile version of `16.04` or greater, the
default is now just to use exit codes for error detection.
---
#### Unrobust Features Removed ([b92074e](https://github.com/galaxyproject/galaxy/commit/b92074e6ff87a19133b4d973577779c4ee6286d7))
A few tool features have ben removed from tools that declare a version of `16.04` or newer.
- The `interepreter=` attribute on `command` blocks has been eliminated. Please use `$\_\_tool\_directory\_\_` from within the tool instead.
- `format="input"` on output datasets has been eliminated, please use `format\_source=` to specify an exact input to derive datatype from.
- Disables extra output file discovery by default, tools must explicitly describe the outputs to collect with `discover\_dataset` tags.
- Tools require a `version` attribute - previously an implicit default to `1.0.0` would be used.
- `$param\_file` has been eliminated.
---
#### Clean Working Directories
Previously, Galaxy would fill tool working directories with files related to
metadata and job metric collection. Tools will no longer be executed in the same directory as these files.
This applies to all tools not just profile `16.04`+ tools.
---
### 16.01
Full [Galaxy changelog](https://docs.galaxyproject.org/en/master/releases/16.01\_announce.html).
---
#### Conda Dependency Resolution ([PR #1345](https://github.com/galaxyproject/galaxy/pull/1345))
```xml
<tool>
...
<requirements>
<requirement type="package" version="0.11.4">FastQC</requirement>
</requirements>
...
</tool>
```
- Dependency resolvers tell Galaxy how to translate requirements into jobs.
- The Conda dependency resolver forces Galaxy to create a conda environment
for the job with `FastQC` at version `0.11.4` installed.
- Only dependency resolver that can be installed at runtime - great for
Docker images, heterogeneous clusters, and testing tools.
- Links [Conda](http://conda.pydata.org/docs/) and [BioConda](https://bioconda.github.io/)
---
#### ToolBox Enhancements - Labels ([PR #1012](https://github.com/galaxyproject/galaxy/pull/1012))
![ToolBox Labels](images/gx\_toolbox\_labels.png)
---
#### ToolBox Enhancements - Monitoring ([PR #1398](https://github.com/galaxyproject/galaxy/pull/1398))
- The Galaxy toolbox can be reloaded from the Admin interface.
- Tool conf files (e.g. `tool\_conf.xml`) can be monitored and automatically
reloaded by Galaxy.
- Tool conf files can now be specified as YAML (or JSON).
---
#### Process Inputs as JSON ([PR #1405](https://github.com/galaxyproject/galaxy/pull/1405))
```xml
<command>python "$\_\_tool\_directory/script.py" "$json\_inputs"</command>
<configfiles>
<inputs name="json\_inputs" />
</configfiles>
```
This will produce a file referenced as `$json\_inputs` that contains a nested
JSON structure corresponding to the tools inputs. Of limitted utility for
simple command-line tools - but complex tools with many repeats, conditional,
and nesting could potentially benefit from this.
For instance, the [JBrowse](https://github.com/galaxyproject/tools-iuc/blob/master/tools/jbrowse/jbrowse.xml)
tool generates a complex JSON data structure using a `configfile` inside the
XML. This is a much more portable way to deal with that.
---
#### Collections
- `data\_collection` tool parameters (`param`s) can now specify multiple
`collection\_type`s for consumption ([PR #1308](https://github.com/galaxyproject/galaxy/pull/1308)).
- This mirrors the `format` attribute which allows a comma-separated list
of potential format types.
- Multiple collections can now be supplied to a `multiple="true"` data parameter ([PR #805](https://github.com/galaxyproject/galaxy/pull/805)).
- Output collections can specify a `type\_source` attribute (again mirroring
`format\_source`) ([PR #1153](https://github.com/galaxyproject/galaxy/pull/1153)).
---
### 15.10
Full [Galaxy changelog](https://docs.galaxyproject.org/en/master/releases/15.10\_announce.html).
---
#### Collections
- Tools may now produce explicit nested outputs [PR #538](https://github.com/galaxyproject/galaxy/pull/538).
This enhances the `discover\_dataset` XML tag to allow this.
- Allow certain `output` actions on collections.
[PR #544](https://github.com/galaxyproject/galaxy/pull/544).
- Allow `discover\_dataset` tags to use `format` instead of `ext`
when referring to datatype extensions/formats.
- Allow `min`/`max` attributes on multiple data input parameters [PR #765](https://github.com/galaxyproject/galaxy/pull/765).
---
#### Whitelist Tools that Generate HTML ([PR #510](https://github.com/galaxyproject/galaxy/pull/510))
Galaxy now contains a plain text file that contains a list of tools whose
output can be trusted when rendering HTML.
---
### 15.07
Full [Galaxy changelog](https://docs.galaxyproject.org/en/master/releases/15.07\_announce.html).
---
#### Parameterized XML Macros ([PR #362](https://github.com/galaxyproject/galaxy/pull/362))
Macros now allow defining tokens to be consumed
as XML attributes. For instance, the following definition
```xml
<tool>
<expand macro="inputs" foo="hello" />
<expand macro="inputs" foo="world" />
<expand macro="inputs" />
<macros>
<xml name="inputs" token\_foo="the\_default">
<inputs>@FOO@</inputs>
</xml>
</macros>
</tool>
```
would expand out as
```xml
<tool>
<inputs>hello</inputs>
<inputs>world</inputs>
<inputs>the\_default</inputs>
</tool>
```
---
#### Tool Form
The workflow editor was updated to the use Galaxy's newer
frontend tool form.
![New Workflow Editor](images/gx\_new\_workflow\_editor.png)
---
#### Environment Variables ([PR #395](https://github.com/galaxyproject/galaxy/pull/395))
Tools may now use `inputs` to define environment variables that will be
set during tool execution. The new `environment\_variables` XML block is
used to define this.
```xml
<command>
echo "\$INTVAR" > $out\_file1;
echo "\$FORTEST" >> $out\_file1;
</command>
<environment\_variables>
<environment\_variable name="INTVAR">$inttest</environment\_variable>
<environment\_variable name="FORTEST">#for i in ['m', 'o', 'o']#$i#end for#</environment\_variable>
</environment\_variables>
...
```
[Test tool](https://github.com/galaxyproject/galaxy/blob/dev/test/functional/tools/environment\_variables.xml) demonstrating the use of the `environment\_variables` tag.
---
#### Collections
- Explicit output collections can now be used in workflows. ([PR #311](https://github.com/galaxyproject/galaxy/pull/311))
- The `filter` tag has been implemented for output dataset collections ([PR #455](https://github.com/galaxyproject/galaxy/pull/455). See the example tool [output\_collection\_filter.xml](https://github.com/galaxyproject/galaxy/blob/dev/test/functional/tools/output\_collection\_filter.xml).
