# Supplementary material for: The Planemo toolkit for developing, deploying, and executing scientific data analyses in Galaxy and beyond
Source: Genome Res. 2023 Feb;33(2):261–8. doi: 10.1101/gr.276963.122 (PMC10069471; doi:10.1101/gr.276963.122)
Supplement: Supplemental Material [file supp_gr.276963.122_Supplemental_Code.tar.gz › planemo-0.75.3/docs/presentations/2016_workflows.html]

Planemo: A Scientific Workflow SDK


class: center, middle
layout: true
class: inverse, middle
---
class: title
# Planemo
## A Scientific Workflow SDK
John Chilton, Aysam Guerler, and the Galaxy Team
The Slides @ http://bit.ly/bosc2016
The Twitters `#usegalaxy` `#commonwl` `@jmchilton`
???
Oh look, I get to follow Björn again - that doesn't seem fair to me.
Compounding that problem - my slides are just the same as last years
Galaxy Tool SDK find and replace Galaxy tool with scientific workflow.
---
class: larger
### A Galaxy Philosophy
\* The most important Galaxy user is the \*bench scientist\* using the GUI, they come first!
\* .smaller[No one wants to inconvenience a bioinformatician or developer, but we will if absolutely required for the biologist.]
\* Galaxy workflows will \*never require an SDK\*.
\* .smaller[Planemo, workflow formats, etc... are conveniences for people who prefer developer processes.]
???
I'm going to get in trouble from a couple different sides with this talk, and I will state some strong, personal opinions. So I'm going to start with something that amounts to either a justification or a reassurance.
I'm pretty confident this amounts to a Galaxy team perspective - regardless
of how your define that team.
READ SLIDES
The first part of this talk will cover workflow enhancements from an end
user perspective in some ways though.
---
class: center
### GUI Enhancements - Workflow Editor Form
![Workflow Editor](images/gx\_new\_workflow\_editor.png)
???
The editor form now uses the same backbone driven MVC components as the
new tool form presented last year.
---
class: center
background-image: url(images/gx\_new\_run\_workflow.png)
background-repeat: no-repeat
background-size: contain
background-position: center
### GUI Enhancements - Workflow Run Form
???
The run workflow form has likewise been overhauled and will be merged soon. This
should allow more dynamic option control when running workflows.
---
class: center
### GUI Enhancements - Labels
![Workflow Output Labels](images/gx\_workflow\_output\_labels.png)
???
When reasoning about workflows and connections between steps, persistent and unique
labels for steps and outputs are important. These are useful in the API and too a
lesser extent in the GUI today.
A major theme of this presentation is going to be that workflows are programs, they
are a coding artifact. I'm not sure anyone would disagree with me on that - but I
think the implications may be counter-intuitive at times.
---
class: center, white
### GUI Enhancements - Nested Workflows
![Nested Workflows](images/gx\_subworkflow\_example\_brad\_langhorst\_neb.png)
Image and workflow thanks to \*Brad Langhorst\* at New England BioLabs.
???
Workflows are programs, languages describing programs should provide abstractions for
composition. Nesting workflows was one of the most requested feature requests of
Galaxy and it now supports this.
---
### What About Planemo?
???
Enough screenshots right - when I present people expect to see long command-lines!
---
### Planemo's Success
.pull-left[
It is \*the way\* to develop Galaxy tools in 2016! Why?
- Artifact-centric - not Galaxy-centric or registry-centric.
- Works with existing developer tools - CLI, Git(hub), CI (Travis).
- Very flexible, easily configurable.
- Well documented with focus on \*usage examples\*.
It is about \*\*developer processes\*\*.
]
.pull-right[
![Nemo](images/nemo.gif)
]
???
In 2014, Greg von Kuster presented a tool development workflow that involved
publishing things to a local tool shed and running tests from there and viewing
the results through the web interface. I call this registry or shed centric tool
development (development activities "boot strap the tool shed, upload to the tool
shed, run tests against the tool shed, view results in the tool shed, export capsule
from the tool shed". Prior to that tool development was Galaxy-centric - "download
Galaxy, update the Galaxy tool conf, update the Galaxy test data, run the Galaxy
tests." Planemo is tool centric - lint the tool, test the tool, serve the tool.
---
class: bottom
background-image: url(images/organic\_mower\_wat.jpg)
background: #FFFFFF
background-repeat: no-repeat
.photo-credit[Photo Credit:
\*Peter Smith (@skwiot)\*]
---
class: larger
### Workflows are Programs
When I write programs...
\* ... I write \*tests\* (and write them first)!
\* ... I commit them to \*Github\*!
\* ... use a text editor - \*my\* text editor!
???
The hippest, artisinal, free range text editor of my choice. It is a go
lang rewrite of a node rewrite of an Erlang editor from 1987 - it is super hot right
now but I'm sure you've never heard of.
---
class: large
### Workflow Operations with Planemo
Serve them:
```
$ planemo serve <workflow.ga>
```
Brings up Galaxy interface with the workflow loaded, install shed
tools as needed.
???
TODO: Install tool shed tools in .ga files, works for format 2 workflows.
---
class: large
### Load up local tools!
Serve them with tools...
```
$ planemo s --extra\_tools <tool\_dir>
<workflow.ga>
```
Supply as many tools as you want.
---
class: larger
### Workflows are harder to serve than tools
\* Longer to setup input data
\* Takes time to install shed tools
\* `sqlite` database locks
\* Cluster access is more important
---
class: large
### Planemo Profiles
Profile - a \*persistent\*, named Galaxy configuration available for serving, running
and testing across workflow invocations.
```
$ planemo profile\_create <name>
$ planemo serve --profile <name> <workflow.ga>
```
---
class: large
### Planemo Profiles and Postgres
```
$ planemo profile\_create --postgres <name>
$ planemo serve --profile <name> <workflow.ga>
```
Automatically \*provision a postgres database\* and configure this profile to use it. All
future `serve`, `run`, and `test` invocations using this profile will use this
database.
???
Makes some reasonable guesses, but Postgres connection settings can be configured with
additional CLI options or in `~/.planemo.yml`.
---
class: large
### Planemo Profiles and Clusters
```
$ planemo profile\_create --job\_conf <job\_conf.xml>
<name>
```
Setup a job configuration object for this profile and use it with all future
`serve`, `run`, and `test` invocations using this profile.
???
TODO: Implement slurm.
---
class: large
### Planemo Docker Profiles
Or just...
.slightly-smaller[```
$ planemo profile\_create --engine\_type docker\_galaxy
<name>
```
]
Leverage the `docker-galaxy-stable` project spearheaded by the
Björn Grüning to `serve` and `run` using Galaxy in a Docker container.
This container comes pre-configured with slurm and Postgres as available locally -
but offers lots of additionals goodies such as Condor, ProFTP, supervisor, and uwsgi.
https://github.com/bgruening/docker-galaxy-stable
???
Leverage the omnipresent `docker-galaxy-stable` community project spearheaded by the
omnipresent Björn Grüning to `serve` and `run` using Galaxy in a Docker container.
---
class: large
### Galaxy Workflow Format
Doesn't Galaxy have a JSON workflow format?
.code[```json
"tool\_state": "{\"\_\_page\_\_\": 0, \"\_\_rerun\_remap\_job\_id\_\_\": null,
\"input1\": \"null\", \"chromInfo\": \"\\\"/home/john/workspace/galaxy-central/tool-data/shared/ucsc/chrom/?.len\\\"\",
\"queries\": \"[{\\\"input2\\\": null, \\\"\_\_index\_\_\\\": 0}]\"}",
```]
- Neither human writable, nor human readable.
- JSON doesn't allow comments.
- One shouldn't have to describe a configuration file in JSON,
let alone write a program in it.
---
class: smaller
### Format 2 Workflows - Example
```yaml
class: GalaxyWorkflow
name: "Test Workflow"
tools:
- name: text\_processing
owner: bgruening
inputs:
- id: input1
outputs:
- id: wf\_output\_1
source: sort#outfile
steps:
- id: sed
tool\_id: tp\_sed\_tool
state:
infile:
$link: input1
code: "s/ World//g"
- id: sort
tool\_id: tp\_sort\_header\_tool
state:
infile:
$link: sed#output
style: h #Human readable
```
Inspired by CWL, Michael Crusoe assures me we can use actual CWL workflow definitions
with Galaxy tool definitions.
---
class: smaller
### Format 2 Workflows - Composition Example
```yaml
class: GalaxyWorkflow
inputs:
- id: outer\_input
steps:
- tool\_id: cat1
label: first\_cat
state:
input1: {$link: outer\_input}
- run:
class: GalaxyWorkflow
inputs:
- id: inner\_input
outputs:
- id: workflow\_output
source: random\_lines#out\_file1
steps:
- tool\_id: random\_lines1
label: random\_lines
state:
num\_lines: 1
input: {$link: inner\_input}
seed\_source:
seed\_source\_selector: set\_seed
seed: asdf
label: nested\_workflow
connect:
inner\_input: first\_cat#out\_file1
...
```
---
class: smaller
### Format 2 Workflows - Implicit Connections Example
```yaml
class: GalaxyWorkflow
name: "Indexing Workflow"
inputs:
- id: fasta
- id: reads
steps:
- label: create\_index
tool\_id: example\_data\_manager
state:
sequences:
$link: fasta
- label: run\_mapper
tool\_id: example\_mapper
connect:
$step: create\_index
state:
input1:
$link: reads
```
---
class: large
### Testing Workflows
```
$ planemo test [--profile <name>] <workflow>
```
\* Same HTML output and other formatting options as tools.
\* Produce sharable test result link with `planemo share\_test`.
\* Test either Galaxy native or Format 2 workflows.
---
class: large
### Generalized Test Format
Test any artifact (Galaxy Tool, Galaxy Workflow, CWL Tool, CWL
Workflow) - using the same YAML-based format.
If workflow is in file `my\_workflow.ga`, place test file named
`my\_workflow-test.yml` in the same directory.
```
planemo test my\_workflow.ga
```
Will detect this artifact and run the tests.
---
class: large, bottom, white
background-image: url(images/tool\_test.png)
background-repeat: no-repeat
background-size: contain
---
```yaml
- doc: Simple test over text tools.
job:
input1:
class: File
path: hello.txt
outputs:
wf\_output\_1:
checksum: sha1$2ef7bde608ce5404e97d5f042f95f89f1c232871
wf\_output\_2:
file: output1.txt
compare: diff
lines\_diff: 2
```
---
class: larger
### Run Workflows
```
$ planemo run <workflow> <job.json>
```
---
class: large, bottom, white
background-image: url(images/CWL-Logo-HD.png)
background-repeat: no-repeat
background-size: contain
---
layout: true
class: inverse, middle
---
class: large
### CWL & Galaxy
\*Experimental\* tool support today using planemo.
```
$ planemo serve --cwl <tool.cwl>
$ planemo test <tool.cwl>
$ planemo run <tool.cwl> <job.json>
```
When `serve`, `test`, `run` encounter CWL tools they will use a Galaxy fork.
Work in progress at https://github.com/common-workflow-language/galaxy.
---
class: large
### Planemo Engine Type `cwltool`
With `--engine\_type=cwltool` (set default in `~/.planemo.yml`), one can
`run` and `test` both CWL tools and workflows.
---
### CWL and `tool\_init` (1 / 2)
```shell
$ planemo tool\_init --cwl \
--id 'seqtk\_seq' \
--name 'Convert to FASTA (seqtk)' \
--example\_command \
'seqtk seq -A 2.fastq > 2.fasta' \
--example\_input 2.fastq \
--example\_output 2.fasta \
--container 'dukegcb/seqtk' \
--test\_case \
--help\_from\_command 'seqtk seq'
```
---
class: smaller
### CWL and `tool\_init` (2 / 2)
.pull-left[
```yaml
#!/usr/bin/env cwl-runner
cwlVersion: 'cwl:draft-3'
class: CommandLineTool
id: "seqtk\_seq"
label: "Convert to FASTA (seqtk)"
requirements:
- class: DockerRequirement
dockerPull: dukegcb/seqtk
inputs:
- id: input1
type: File
description: TODO
inputBinding:
position: 1
prefix: "-a"
outputs:
- id: output1
type: File
outputBinding:
glob: out
baseCommand: ["seqtk", "seq"]
arguments: []
stdout: out
description: |
Usage: seqtk seq [options] <in.fq>|<in.fa>
...
```
]
.large[.pull-right[
Generates:
- `seqtk\_seq.cwl`
- `seqtk\_seq-tests.yml`
- `test-data/2.fasta`
- `test-data/2.fastq`
]]
---
### CWL and `lint`
```
$ planemo l seqtk\_seq.cwl
Linting tool /opt/tools/seqtk\_seq.cwl
Applying linter general... CHECK
.. CHECK: Tool defines a version [0.0.1].
.. CHECK: Tool defines a name [Convert to FASTA (seqtk)].
.. CHECK: Tool defines an id [seqtk\_seq\_v3].
Applying linter cwl\_validation... CHECK
.. INFO: CWL appears to be valid.
Applying linter docker\_image... CHECK
.. INFO: Tool will run in Docker image [dukegcb/seqtk].
Applying linter new\_draft... CHECK
.. INFO: Modern CWL version [cwl:draft-3]
```
---
class: large
### CWL and `test`
```
$ planemo t --engine\_type=cwltool \
seqtk\_seq.cwl
```
Tool test output HTML is produced in the file `tool\_test\_output.html`.
---
class: large
### Deployment Matters
- `SoftwareRequirement`s were added to CWL 1.0. [PR #240](https://github.com/common-workflow-language/common-workflow-language/pull/240)
- Can be resolved using Galaxy's dependency resolution framework - [cwltool#93](https://github.com/common-workflow-language/cwltool/pull/93)
- Conda, homebrew, environment modules, "galaxy packages", etc....
---
class: large, bottom, white
background-image: url(images/mulledflow.png)
background-repeat: no-repeat
background-size: contain
---
class: inverse
background-color: black
### Thanks
.pull-left[
- \*Planemo contributors\*
- Galaxy team & IUC
- Common Workflow Language group - with special thanks to \*Michael Crusoe\* and \*Peter Amstutz\* for working through detailed compromises
- Everyone that has ever built something cool with Planemo or Galaxy workflows
]
.smaller.pull-right[- \*Helena Rasche\*
- \*Martin Cech\*
- \*Peter Cock\*
- \*Daniel Blankenberg\*
- \*Björn Grüning\*
- \*Dave Bouvier\*
- \*Kyle Ellrott\*
- \*Nate Coraor\*
- \*Dannon Baker\*
- \*Marius van den Beek\*
- \*Lance Parsons\*
- \*Nicola Soranzo\*
- \*Dannon Baker\*
- \*James Taylor\*
- \*Mark Einon\*
- \*Michael R. Crusoe\*
- \*Peter van Heusden\*
- \*Rémi Marenco\*
- \*Matt Chambers\*
- \*Gildas Le Corguillé\*
- \*Nitesh Turaga\*
]
---
class: large
### Aside - gxformat2
```
pip install gxformat2
```
`gxformat2` is a Python library for the conversion of "Format 2" workflows.
- Started as a way to build test workflows for Galaxy testing framework.
- All steps can be labeled, connections described by ID.
- Pypi @ https://pypi.python.org/pypi/gxformat2/
- Github @ https://github.com/jmchilton/gxformat2
---
class: large
### Another Aside - Ephemeris
```
pip install emphemeris
```
`emphemeris` is an opinionated Python library and scripts for bootstrapping Galaxy tools, index data, and worklows.
- Scripts from `ansible-galaxy-tools` by Marius van den Beek, Enis Afgane, Björn Grüning, and others.
- Pypi @ https://pypi.python.org/pypi/emphemeris/
- Github @ https://github.com/galaxyproject/emphemeris
---
class: large
### Common Workflow Language
\* http://www.commonwl.org/
\* Group of (and specifications by) engineers
\* Formed at 2014 BOSC Codefest.
\* After 4 draft iterations, 1.0 will be released next week.
\* Like Galaxy, the rare open infrastructure that crosses the Atlantic.
\* Adopted by various Elixer efforts, Seven Bridge Genomics,
and will be implemented in Taverna.
\* Will be supported on all NIH Cancer Cloud Pilots and endorsed by the
GA4GH. Reference implementation `cwltool` developed at Curoverse.
