# Supplementary material for: The Planemo toolkit for developing, deploying, and executing scientific data analyses in Galaxy and beyond
Source: Genome Res. 2023 Feb;33(2):261–8. doi: 10.1101/gr.276963.122 (PMC10069471; doi:10.1101/gr.276963.122)
Supplement: Supplemental Material [file supp_gr.276963.122_Supplemental_Code.tar.gz › planemo-0.75.3/planemo/reports/report_html.tpl]

{{ title }}

{{ bootstrap\_style }}
{{ custom\_style }}


Toggle navigation

{{ title }}

- Galaxy
- Planemo

{{ jquery\_script }}
{{ bootstrap\_script }}
{{ markdown\_it\_script }}
