# Supplementary material for: The Planemo toolkit for developing, deploying, and executing scientific data analyses in Galaxy and beyond
Source: Genome Res. 2023 Feb;33(2):261–8. doi: 10.1101/gr.276963.122 (PMC10069471; doi:10.1101/gr.276963.122)
Supplement: Supplemental Material [file supp_gr.276963.122_Supplemental_Code.tar.gz › planemo-0.75.3/tests/data/tt_fail.html]

ID: functional.test\_toolbox.TestForTool\_cat.test\_tool\_000000

Description: Concatenate datasets (for test workflows) ( cat ) > Test-1

Status: failure

Log: ...

```
requests.packages.urllib3.connectionpool: INFO: Starting new HTTP connection (1): localhost
galaxy.web.framework.webapp: INFO: Session authenticated using Galaxy master api key
requests.packages.urllib3.connectionpool: DEBUG: "GET /api/users?key=test_key HTTP/1.1" 200 None
requests.packages.urllib3.connectionpool: INFO: Starting new HTTP connection (1): localhost
galaxy.web.framework.webapp: INFO: Session authenticated using Galaxy master api key
requests.packages.urllib3.connectionpool: DEBUG: "POST /api/users HTTP/1.1" 200 None
requests.packages.urllib3.connectionpool: INFO: Starting new HTTP connection (1): localhost
galaxy.web.framework.webapp: INFO: Session authenticated using Galaxy master api key
requests.packages.urllib3.connectionpool: DEBUG: "POST /api/users/5729865256bc2525/api_key HTTP/1.1" 200 None
requests.packages.urllib3.connectionpool: INFO: Starting new HTTP connection (1): localhost
requests.packages.urllib3.connectionpool: DEBUG: "POST /api/histories HTTP/1.1" 200 None
requests.packages.urllib3.connectionpool: INFO: Starting new HTTP connection (1): localhost
galaxy.tools: DEBUG: Validated and populated state for tool request (41.843 ms)
galaxy.tools.actions.upload: DEBUG: Persisted uploads (20.623 ms)
galaxy.tools.actions.upload: DEBUG: Checked and cleaned uploads (448.852 ms)
galaxy.tools.actions.upload_common: INFO: tool upload1 created job id 1
galaxy.tools.actions.upload: DEBUG: Created upload job (225.630 ms)
galaxy.tools.execute: DEBUG: Tool [upload1] created job [1] (695.995 ms)
galaxy.tools.execute: DEBUG: Executed 1 job(s) for tool upload1 request: (734.929 ms)
requests.packages.urllib3.connectionpool: DEBUG: "POST /api/tools HTTP/1.1" 200 None
requests.packages.urllib3.connectionpool: INFO: Starting new HTTP connection (1): localhost
galaxy.jobs: DEBUG: (1) Working directory for job is: /tmp/tmpwwBgVs/job_working_directory/000/1
requests.packages.urllib3.connectionpool: DEBUG: "GET /api/histories/2891970512fa2d5a?key=5723299c7bc15b8cb8b00e98a08e9203 HTTP/1.1" 200 None
requests.packages.urllib3.connectionpool: INFO: Starting new HTTP connection (1): localhost
galaxy.jobs.handler: DEBUG: (1) Dispatching to local runner
galaxy.jobs: DEBUG: (1) Persisting job destination (destination id: local:///)
galaxy.jobs.runners: DEBUG: Job [1] queued (129.914 ms)
galaxy.jobs.handler: INFO: (1) Job dispatched
requests.packages.urllib3.connectionpool: DEBUG: "GET /api/histories/2891970512fa2d5a?key=5723299c7bc15b8cb8b00e98a08e9203 HTTP/1.1" 200 None
requests.packages.urllib3.connectionpool: INFO: Starting new HTTP connection (1): localhost
requests.packages.urllib3.connectionpool: DEBUG: "GET /api/histories/2891970512fa2d5a?key=5723299c7bc15b8cb8b00e98a08e9203 HTTP/1.1" 200 None
requests.packages.urllib3.connectionpool: INFO: Starting new HTTP connection (1): localhost
galaxy.tools.deps: DEBUG: Building dependency shell command for dependency 'samtools'
galaxy.tools.deps: DEBUG: Find dependency samtools version None
galaxy.tools.deps: WARNING: Failed to resolve dependency on 'samtools', ignoring
requests.packages.urllib3.connectionpool: DEBUG: "GET /api/histories/2891970512fa2d5a?key=5723299c7bc15b8cb8b00e98a08e9203 HTTP/1.1" 200 None
requests.packages.urllib3.connectionpool: INFO: Starting new HTTP connection (1): localhost
galaxy.jobs.command_factory: INFO: Built script [/tmp/tmpwwBgVs/job_working_directory/000/1/tool_script.sh] for tool command[python /home/john/workspace/galaxy/tools/data_source/upload.py /home/john/workspace/galaxy /tmp/tmpwwBgVs/tmp/tmpeSjJLn /tmp/tmpwwBgVs/tmp/tmplvJZM6 1:/tmp/tmpwwBgVs/job_working_directory/000/1/dataset_1_files:/tmp/tmpwwBgVs/files/000/dataset_1.dat]
requests.packages.urllib3.connectionpool: DEBUG: "GET /api/histories/2891970512fa2d5a?key=5723299c7bc15b8cb8b00e98a08e9203 HTTP/1.1" 200 None
requests.packages.urllib3.connectionpool: INFO: Starting new HTTP connection (1): localhost
galaxy.tools.deps: DEBUG: Building dependency shell command for dependency 'samtools'
galaxy.tools.deps: DEBUG: Find dependency samtools version None
galaxy.tools.deps: WARNING: Failed to resolve dependency on 'samtools', ignoring
galaxy.jobs.runners: DEBUG: (1) command is: mkdir -p working; cd working; /tmp/tmpwwBgVs/job_working_directory/000/1/tool_script.sh; return_code=$?; cd '/tmp/tmpwwBgVs/job_working_directory/000/1'; python "/tmp/tmpwwBgVs/job_working_directory/000/1/set_metadata_2Ah3js.py" "/tmp/tmpwwBgVs/tmp/tmpeSjJLn" "/tmp/tmpwwBgVs/job_working_directory/000/1/working/galaxy.json" "/tmp/tmpwwBgVs/job_working_directory/000/1/metadata_in_HistoryDatasetAssociation_1_RXN9pp,/tmp/tmpwwBgVs/job_working_directory/000/1/metadata_kwds_HistoryDatasetAssociation_1_uz8gO5,/tmp/tmpwwBgVs/job_working_directory/000/1/metadata_out_HistoryDatasetAssociation_1_mG60zC,/tmp/tmpwwBgVs/job_working_directory/000/1/metadata_results_HistoryDatasetAssociation_1_6Lkep2,/tmp/tmpwwBgVs/files/000/dataset_1.dat,/tmp/tmpwwBgVs/job_working_directory/000/1/metadata_override_HistoryDatasetAssociation_1_RIXczB" 5242880; sh -c "exit $return_code"
galaxy.jobs.runners.local: DEBUG: (1) executing job script: /tmp/tmpwwBgVs/job_working_directory/000/1/galaxy_1.sh
galaxy.jobs: DEBUG: (1) Persisting job destination (destination id: local:///)
requests.packages.urllib3.connectionpool: DEBUG: "GET /api/histories/2891970512fa2d5a?key=5723299c7bc15b8cb8b00e98a08e9203 HTTP/1.1" 200 None
requests.packages.urllib3.connectionpool: INFO: Starting new HTTP connection (1): localhost
galaxy.jobs.runners.local: DEBUG: execution finished: /tmp/tmpwwBgVs/job_working_directory/000/1/galaxy_1.sh
requests.packages.urllib3.connectionpool: DEBUG: "GET /api/histories/2891970512fa2d5a?key=5723299c7bc15b8cb8b00e98a08e9203 HTTP/1.1" 200 None
requests.packages.urllib3.connectionpool: INFO: Starting new HTTP connection (1): localhost
galaxy.model.metadata: DEBUG: loading metadata from file for: HistoryDatasetAssociation 1
requests.packages.urllib3.connectionpool: DEBUG: "GET /api/histories/2891970512fa2d5a?key=5723299c7bc15b8cb8b00e98a08e9203 HTTP/1.1" 200 None
requests.packages.urllib3.connectionpool: INFO: Starting new HTTP connection (1): localhost
galaxy.tools: DEBUG: Validated and populated state for tool request (114.852 ms)
galaxy.jobs: DEBUG: job 1 ended (finish() executed in (753.726 ms))
galaxy.tools.actions: INFO: Handled output named out_file1 for tool cat (192.326 ms)
galaxy.tools.actions: INFO: Added output datasets to history (62.733 ms)
galaxy.tools.actions: INFO: Verified access to datasets for Job[unflushed,tool_id=cat] (12.053 ms)
galaxy.tools.actions: INFO: Setup for job Job[unflushed,tool_id=cat] complete, ready to flush (30.756 ms)
galaxy.tools.actions: INFO: Flushed transaction for job Job[id=2,tool_id=cat] (72.908 ms)
galaxy.tools.execute: DEBUG: Tool [cat] created job [2] (380.756 ms)
galaxy.tools.execute: DEBUG: Executed 1 job(s) for tool cat request: (440.672 ms)
requests.packages.urllib3.connectionpool: DEBUG: "POST /api/tools HTTP/1.1" 200 None
requests.packages.urllib3.connectionpool: INFO: Starting new HTTP connection (1): localhost
requests.packages.urllib3.connectionpool: DEBUG: "GET /api/jobs/5729865256bc2525?key=5723299c7bc15b8cb8b00e98a08e9203 HTTP/1.1" 200 None
requests.packages.urllib3.connectionpool: INFO: Starting new HTTP connection (1): localhost
requests.packages.urllib3.connectionpool: DEBUG: "GET /api/jobs/5729865256bc2525?key=5723299c7bc15b8cb8b00e98a08e9203 HTTP/1.1" 200 None
galaxy.jobs: DEBUG: (2) Working directory for job is: /tmp/tmpwwBgVs/job_working_directory/000/2
galaxy.jobs.handler: DEBUG: (2) Dispatching to local runner
galaxy.jobs: DEBUG: (2) Persisting job destination (destination id: local:///)
requests.packages.urllib3.connectionpool: INFO: Starting new HTTP connection (1): localhost
galaxy.jobs.runners: DEBUG: Job [2] queued (121.122 ms)
galaxy.jobs.handler: INFO: (2) Job dispatched
requests.packages.urllib3.connectionpool: DEBUG: "GET /api/jobs/5729865256bc2525?key=5723299c7bc15b8cb8b00e98a08e9203 HTTP/1.1" 200 None
galaxy.jobs.command_factory: INFO: Built script [/tmp/tmpwwBgVs/job_working_directory/000/2/tool_script.sh] for tool command[cat /tmp/tmpwwBgVs/files/000/dataset_1.dat  > /tmp/tmpwwBgVs/files/000/dataset_2.dat]
galaxy.tools.deps: DEBUG: Building dependency shell command for dependency 'samtools'
galaxy.tools.deps: DEBUG: Find dependency samtools version None
galaxy.tools.deps: WARNING: Failed to resolve dependency on 'samtools', ignoring
galaxy.jobs.runners: DEBUG: (2) command is: mkdir -p working; cd working; /tmp/tmpwwBgVs/job_working_directory/000/2/tool_script.sh; return_code=$?; cd '/tmp/tmpwwBgVs/job_working_directory/000/2'; python "/tmp/tmpwwBgVs/job_working_directory/000/2/set_metadata_gj6rLF.py" "/tmp/tmpwwBgVs/tmp/tmpeSjJLn" "/tmp/tmpwwBgVs/job_working_directory/000/2/working/galaxy.json" "/tmp/tmpwwBgVs/job_working_directory/000/2/metadata_in_HistoryDatasetAssociation_2_xyfvRc,/tmp/tmpwwBgVs/job_working_directory/000/2/metadata_kwds_HistoryDatasetAssociation_2_7VahWW,/tmp/tmpwwBgVs/job_working_directory/000/2/metadata_out_HistoryDatasetAssociation_2_SiN6cX,/tmp/tmpwwBgVs/job_working_directory/000/2/metadata_results_HistoryDatasetAssociation_2_Us0uvr,/tmp/tmpwwBgVs/files/000/dataset_2.dat,/tmp/tmpwwBgVs/job_working_directory/000/2/metadata_override_HistoryDatasetAssociation_2_mYUpFh" 5242880; sh -c "exit $return_code"
galaxy.jobs.runners.local: DEBUG: (2) executing job script: /tmp/tmpwwBgVs/job_working_directory/000/2/galaxy_2.sh
galaxy.jobs: DEBUG: (2) Persisting job destination (destination id: local:///)
requests.packages.urllib3.connectionpool: INFO: Starting new HTTP connection (1): localhost
requests.packages.urllib3.connectionpool: DEBUG: "GET /api/jobs/5729865256bc2525?key=5723299c7bc15b8cb8b00e98a08e9203 HTTP/1.1" 200 None
galaxy.jobs.runners.local: DEBUG: execution finished: /tmp/tmpwwBgVs/job_working_directory/000/2/galaxy_2.sh
galaxy.model.metadata: DEBUG: loading metadata from file for: HistoryDatasetAssociation 2
requests.packages.urllib3.connectionpool: INFO: Starting new HTTP connection (1): localhost
requests.packages.urllib3.connectionpool: DEBUG: "GET /api/jobs/5729865256bc2525?key=5723299c7bc15b8cb8b00e98a08e9203 HTTP/1.1" 200 None
galaxy.jobs: DEBUG: job 2 ended (finish() executed in (619.008 ms))
requests.packages.urllib3.connectionpool: INFO: Starting new HTTP connection (1): localhost
requests.packages.urllib3.connectionpool: DEBUG: "GET /api/jobs/5729865256bc2525?key=5723299c7bc15b8cb8b00e98a08e9203 HTTP/1.1" 200 None
requests.packages.urllib3.connectionpool: INFO: Starting new HTTP connection (1): localhost
requests.packages.urllib3.connectionpool: DEBUG: "GET /api/jobs/5729865256bc2525?full=true&key=5723299c7bc15b8cb8b00e98a08e9203 HTTP/1.1" 200 None
requests.packages.urllib3.connectionpool: INFO: Starting new HTTP connection (1): localhost
requests.packages.urllib3.connectionpool: DEBUG: "GET /api/jobs/5729865256bc2525?key=5723299c7bc15b8cb8b00e98a08e9203 HTTP/1.1" 200 None
requests.packages.urllib3.connectionpool: INFO: Starting new HTTP connection (1): localhost
requests.packages.urllib3.connectionpool: DEBUG: "GET /api/histories/2891970512fa2d5a/contents/5729865256bc2525/display?raw=true&key=5723299c7bc15b8cb8b00e98a08e9203 HTTP/1.1" 200 None
```

Exception: ...

```
Traceback (most recent call last):

  File "/usr/lib/python2.7/unittest/case.py", line 331, in run
    testMethod()

  File "/home/john/workspace/galaxy/test/functional/test_toolbox.py", line 299, in test_tool
    self.do_it( td )

  File "/home/john/workspace/galaxy/test/functional/test_toolbox.py", line 75, in do_it
    raise e

JobOutputsError: History item  different than expected
No line of output file was 'MooCow' (output was 'chr1	147962192	147962580	CCDS989.1_cds_0_0_chr1_147962193_r	0	-
chr1	147984545	147984630	CCDS990.1_cds_0_0_chr1_147984546_f	0	+
chr1	148078400	148078582	CCDS993.1_cds_0_0_chr1_148078401_r	0	-
chr1	148185136	148185276	CCDS996.1_cds_0_0_chr1_148185137_f	0	+
chr10	55251623	55253124	CCDS7248.1_cds_0_0_chr10_55251624_r	0	-
chr11	116124407	116124501	CCDS8374.1_cds_0_0_chr11_116124408_r	0	-
chr11	116206508	116206563	CCDS8377.1_cds_0_0_chr11_116206509_f	0	+
chr11	116211733	116212337	CCDS8378.1_cds_0_0_chr11_116211734_r	0	-
chr11	1812377	1812407	CCDS7726.1_cds_0_0_chr11_1812378_f	0	+
chr12	38440094	38440321	CCDS8736.1_cds_0_0_chr12_38440095_r	0	-
chr13	112381694	112381953	CCDS9526.1_cds_0_0_chr13_112381695_f	0	+
chr14	98710240	98712285	CCDS9949.1_cds_0_0_chr14_98710241_r	0	-
chr15	41486872	41487060	CCDS10096.1_cds_0_0_chr15_41486873_r	0	-
chr15	41673708	41673857	CCDS10097.1_cds_0_0_chr15_41673709_f	0	+
chr15	41679161	41679250	CCDS10098.1_cds_0_0_chr15_41679162_r	0	-
chr15	41826029	41826196	CCDS10101.1_cds_0_0_chr15_41826030_f	0	+
chr16	142908	143003	CCDS10397.1_cds_0_0_chr16_142909_f	0	+
chr16	179963	180135	CCDS10401.1_cds_0_0_chr16_179964_r	0	-
chr16	244413	244681	CCDS10402.1_cds_0_0_chr16_244414_f	0	+
chr16	259268	259383	CCDS10403.1_cds_0_0_chr16_259269_r	0	-
chr18	23786114	23786321	CCDS11891.1_cds_0_0_chr18_23786115_r	0	-
chr18	59406881	59407046	CCDS11985.1_cds_0_0_chr18_59406882_f	0	+
chr18	59455932	59456337	CCDS11986.1_cds_0_0_chr18_59455933_r	0	-
chr18	59600586	59600754	CCDS11988.1_cds_0_0_chr18_59600587_f	0	+
chr19	59068595	59069564	CCDS12866.1_cds_0_0_chr19_59068596_f	0	+
chr19	59236026	59236146	CCDS12872.1_cds_0_0_chr19_59236027_r	0	-
chr19	59297998	59298008	CCDS12877.1_cds_0_0_chr19_59297999_f	0	+
chr19	59302168	59302288	CCDS12878.1_cds_0_0_chr19_59302169_r	0	-
chr2	118288583	118288668	CCDS2120.1_cds_0_0_chr2_118288584_f	0	+
chr2	118394148	118394202	CCDS2121.1_cds_0_0_chr2_118394149_r	0	-
chr2	220190202	220190242	CCDS2441.1_cds_0_0_chr2_220190203_f	0	+
chr2	220229609	220230869	CCDS2443.1_cds_0_0_chr2_220229610_r	0	-
chr20	33330413	33330423	CCDS13249.1_cds_0_0_chr20_33330414_r	0	-
chr20	33513606	33513792	CCDS13255.1_cds_0_0_chr20_33513607_f	0	+
chr20	33579500	33579527	CCDS13256.1_cds_0_0_chr20_33579501_r	0	-
chr20	33593260	33593348	CCDS13257.1_cds_0_0_chr20_33593261_f	0	+
chr21	32707032	32707192	CCDS13614.1_cds_0_0_chr21_32707033_f	0	+
chr21	32869641	32870022	CCDS13615.1_cds_0_0_chr21_32869642_r	0	-
chr21	33321040	33322012	CCDS13620.1_cds_0_0_chr21_33321041_f	0	+
chr21	33744994	33745040	CCDS13625.1_cds_0_0_chr21_33744995_r	0	-
chr22	30120223	30120265	CCDS13897.1_cds_0_0_chr22_30120224_f	0	+
chr22	30160419	30160661	CCDS13898.1_cds_0_0_chr22_30160420_r	0	-
chr22	30665273	30665360	CCDS13901.1_cds_0_0_chr22_30665274_f	0	+
chr22	30939054	30939266	CCDS13903.1_cds_0_0_chr22_30939055_r	0	-
chr5	131424298	131424460	CCDS4149.1_cds_0_0_chr5_131424299_f	0	+
chr5	131556601	131556672	CCDS4151.1_cds_0_0_chr5_131556602_r	0	-
chr5	131621326	131621419	CCDS4152.1_cds_0_0_chr5_131621327_f	0	+
chr5	131847541	131847666	CCDS4155.1_cds_0_0_chr5_131847542_r	0	-
chr6	108299600	108299744	CCDS5061.1_cds_0_0_chr6_108299601_r	0	-
chr6	108594662	108594687	CCDS5063.1_cds_0_0_chr6_108594663_f	0	+
chr6	108640045	108640151	CCDS5064.1_cds_0_0_chr6_108640046_r	0	-
chr6	108722976	108723115	CCDS5067.1_cds_0_0_chr6_108722977_f	0	+
chr7	113660517	113660685	CCDS5760.1_cds_0_0_chr7_113660518_f	0	+
chr7	116512159	116512389	CCDS5771.1_cds_0_0_chr7_116512160_r	0	-
chr7	116714099	116714152	CCDS5773.1_cds_0_0_chr7_116714100_f	0	+
chr7	116945541	116945787	CCDS5774.1_cds_0_0_chr7_116945542_r	0	-
chr8	118881131	118881317	CCDS6324.1_cds_0_0_chr8_118881132_r	0	-
chr9	128764156	128764189	CCDS6914.1_cds_0_0_chr9_128764157_f	0	+
chr9	128787519	128789136	CCDS6915.1_cds_0_0_chr9_128787520_r	0	-
chr9	128882427	128882523	CCDS6917.1_cds_0_0_chr9_128882428_f	0	+
chr9	128937229	128937445	CCDS6919.1_cds_0_0_chr9_128937230_r	0	-
chrX	122745047	122745924	CCDS14606.1_cds_0_0_chrX_122745048_f	0	+
chrX	152648964	152649196	CCDS14733.1_cds_0_0_chrX_152648965_r	0	-
chrX	152691446	152691471	CCDS14735.1_cds_0_0_chrX_152691447_f	0	+
chrX	152694029	152694263	CCDS14736.1_cds_0_0_chrX_152694030_r	0	-
') 
-------------------- >> begin captured logging << --------------------
requests.packages.urllib3.connectionpool: INFO: Starting new HTTP connection (1): localhost
galaxy.web.framework.webapp: INFO: Session authenticated using Galaxy master api key
requests.packages.urllib3.connectionpool: DEBUG: "GET /api/users?key=test_key HTTP/1.1" 200 None
requests.packages.urllib3.connectionpool: INFO: Starting new HTTP connection (1): localhost
galaxy.web.framework.webapp: INFO: Session authenticated using Galaxy master api key
requests.packages.urllib3.connectionpool: DEBUG: "POST /api/users HTTP/1.1" 200 None
requests.packages.urllib3.connectionpool: INFO: Starting new HTTP connection (1): localhost
galaxy.web.framework.webapp: INFO: Session authenticated using Galaxy master api key
requests.packages.urllib3.connectionpool: DEBUG: "POST /api/users/5729865256bc2525/api_key HTTP/1.1" 200 None
requests.packages.urllib3.connectionpool: INFO: Starting new HTTP connection (1): localhost
requests.packages.urllib3.connectionpool: DEBUG: "POST /api/histories HTTP/1.1" 200 None
requests.packages.urllib3.connectionpool: INFO: Starting new HTTP connection (1): localhost
galaxy.tools: DEBUG: Validated and populated state for tool request (41.843 ms)
galaxy.tools.actions.upload: DEBUG: Persisted uploads (20.623 ms)
galaxy.tools.actions.upload: DEBUG: Checked and cleaned uploads (448.852 ms)
galaxy.tools.actions.upload_common: INFO: tool upload1 created job id 1
galaxy.tools.actions.upload: DEBUG: Created upload job (225.630 ms)
galaxy.tools.execute: DEBUG: Tool [upload1] created job [1] (695.995 ms)
galaxy.tools.execute: DEBUG: Executed 1 job(s) for tool upload1 request: (734.929 ms)
requests.packages.urllib3.connectionpool: DEBUG: "POST /api/tools HTTP/1.1" 200 None
requests.packages.urllib3.connectionpool: INFO: Starting new HTTP connection (1): localhost
galaxy.jobs: DEBUG: (1) Working directory for job is: /tmp/tmpwwBgVs/job_working_directory/000/1
requests.packages.urllib3.connectionpool: DEBUG: "GET /api/histories/2891970512fa2d5a?key=5723299c7bc15b8cb8b00e98a08e9203 HTTP/1.1" 200 None
requests.packages.urllib3.connectionpool: INFO: Starting new HTTP connection (1): localhost
galaxy.jobs.handler: DEBUG: (1) Dispatching to local runner
galaxy.jobs: DEBUG: (1) Persisting job destination (destination id: local:///)
galaxy.jobs.runners: DEBUG: Job [1] queued (129.914 ms)
galaxy.jobs.handler: INFO: (1) Job dispatched
requests.packages.urllib3.connectionpool: DEBUG: "GET /api/histories/2891970512fa2d5a?key=5723299c7bc15b8cb8b00e98a08e9203 HTTP/1.1" 200 None
requests.packages.urllib3.connectionpool: INFO: Starting new HTTP connection (1): localhost
requests.packages.urllib3.connectionpool: DEBUG: "GET /api/histories/2891970512fa2d5a?key=5723299c7bc15b8cb8b00e98a08e9203 HTTP/1.1" 200 None
requests.packages.urllib3.connectionpool: INFO: Starting new HTTP connection (1): localhost
galaxy.tools.deps: DEBUG: Building dependency shell command for dependency 'samtools'
galaxy.tools.deps: DEBUG: Find dependency samtools version None
galaxy.tools.deps: WARNING: Failed to resolve dependency on 'samtools', ignoring
requests.packages.urllib3.connectionpool: DEBUG: "GET /api/histories/2891970512fa2d5a?key=5723299c7bc15b8cb8b00e98a08e9203 HTTP/1.1" 200 None
requests.packages.urllib3.connectionpool: INFO: Starting new HTTP connection (1): localhost
galaxy.jobs.command_factory: INFO: Built script [/tmp/tmpwwBgVs/job_working_directory/000/1/tool_script.sh] for tool command[python /home/john/workspace/galaxy/tools/data_source/upload.py /home/john/workspace/galaxy /tmp/tmpwwBgVs/tmp/tmpeSjJLn /tmp/tmpwwBgVs/tmp/tmplvJZM6 1:/tmp/tmpwwBgVs/job_working_directory/000/1/dataset_1_files:/tmp/tmpwwBgVs/files/000/dataset_1.dat]
requests.packages.urllib3.connectionpool: DEBUG: "GET /api/histories/2891970512fa2d5a?key=5723299c7bc15b8cb8b00e98a08e9203 HTTP/1.1" 200 None
requests.packages.urllib3.connectionpool: INFO: Starting new HTTP connection (1): localhost
galaxy.tools.deps: DEBUG: Building dependency shell command for dependency 'samtools'
galaxy.tools.deps: DEBUG: Find dependency samtools version None
galaxy.tools.deps: WARNING: Failed to resolve dependency on 'samtools', ignoring
galaxy.jobs.runners: DEBUG: (1) command is: mkdir -p working; cd working; /tmp/tmpwwBgVs/job_working_directory/000/1/tool_script.sh; return_code=$?; cd '/tmp/tmpwwBgVs/job_working_directory/000/1'; python "/tmp/tmpwwBgVs/job_working_directory/000/1/set_metadata_2Ah3js.py" "/tmp/tmpwwBgVs/tmp/tmpeSjJLn" "/tmp/tmpwwBgVs/job_working_directory/000/1/working/galaxy.json" "/tmp/tmpwwBgVs/job_working_directory/000/1/metadata_in_HistoryDatasetAssociation_1_RXN9pp,/tmp/tmpwwBgVs/job_working_directory/000/1/metadata_kwds_HistoryDatasetAssociation_1_uz8gO5,/tmp/tmpwwBgVs/job_working_directory/000/1/metadata_out_HistoryDatasetAssociation_1_mG60zC,/tmp/tmpwwBgVs/job_working_directory/000/1/metadata_results_HistoryDatasetAssociation_1_6Lkep2,/tmp/tmpwwBgVs/files/000/dataset_1.dat,/tmp/tmpwwBgVs/job_working_directory/000/1/metadata_override_HistoryDatasetAssociation_1_RIXczB" 5242880; sh -c "exit $return_code"
galaxy.jobs.runners.local: DEBUG: (1) executing job script: /tmp/tmpwwBgVs/job_working_directory/000/1/galaxy_1.sh
galaxy.jobs: DEBUG: (1) Persisting job destination (destination id: local:///)
requests.packages.urllib3.connectionpool: DEBUG: "GET /api/histories/2891970512fa2d5a?key=5723299c7bc15b8cb8b00e98a08e9203 HTTP/1.1" 200 None
requests.packages.urllib3.connectionpool: INFO: Starting new HTTP connection (1): localhost
galaxy.jobs.runners.local: DEBUG: execution finished: /tmp/tmpwwBgVs/job_working_directory/000/1/galaxy_1.sh
requests.packages.urllib3.connectionpool: DEBUG: "GET /api/histories/2891970512fa2d5a?key=5723299c7bc15b8cb8b00e98a08e9203 HTTP/1.1" 200 None
requests.packages.urllib3.connectionpool: INFO: Starting new HTTP connection (1): localhost
galaxy.model.metadata: DEBUG: loading metadata from file for: HistoryDatasetAssociation 1
requests.packages.urllib3.connectionpool: DEBUG: "GET /api/histories/2891970512fa2d5a?key=5723299c7bc15b8cb8b00e98a08e9203 HTTP/1.1" 200 None
requests.packages.urllib3.connectionpool: INFO: Starting new HTTP connection (1): localhost
galaxy.tools: DEBUG: Validated and populated state for tool request (114.852 ms)
galaxy.jobs: DEBUG: job 1 ended (finish() executed in (753.726 ms))
galaxy.tools.actions: INFO: Handled output named out_file1 for tool cat (192.326 ms)
galaxy.tools.actions: INFO: Added output datasets to history (62.733 ms)
galaxy.tools.actions: INFO: Verified access to datasets for Job[unflushed,tool_id=cat] (12.053 ms)
galaxy.tools.actions: INFO: Setup for job Job[unflushed,tool_id=cat] complete, ready to flush (30.756 ms)
galaxy.tools.actions: INFO: Flushed transaction for job Job[id=2,tool_id=cat] (72.908 ms)
galaxy.tools.execute: DEBUG: Tool [cat] created job [2] (380.756 ms)
galaxy.tools.execute: DEBUG: Executed 1 job(s) for tool cat request: (440.672 ms)
requests.packages.urllib3.connectionpool: DEBUG: "POST /api/tools HTTP/1.1" 200 None
requests.packages.urllib3.connectionpool: INFO: Starting new HTTP connection (1): localhost
requests.packages.urllib3.connectionpool: DEBUG: "GET /api/jobs/5729865256bc2525?key=5723299c7bc15b8cb8b00e98a08e9203 HTTP/1.1" 200 None
requests.packages.urllib3.connectionpool: INFO: Starting new HTTP connection (1): localhost
requests.packages.urllib3.connectionpool: DEBUG: "GET /api/jobs/5729865256bc2525?key=5723299c7bc15b8cb8b00e98a08e9203 HTTP/1.1" 200 None
galaxy.jobs: DEBUG: (2) Working directory for job is: /tmp/tmpwwBgVs/job_working_directory/000/2
galaxy.jobs.handler: DEBUG: (2) Dispatching to local runner
galaxy.jobs: DEBUG: (2) Persisting job destination (destination id: local:///)
requests.packages.urllib3.connectionpool: INFO: Starting new HTTP connection (1): localhost
galaxy.jobs.runners: DEBUG: Job [2] queued (121.122 ms)
galaxy.jobs.handler: INFO: (2) Job dispatched
requests.packages.urllib3.connectionpool: DEBUG: "GET /api/jobs/5729865256bc2525?key=5723299c7bc15b8cb8b00e98a08e9203 HTTP/1.1" 200 None
galaxy.jobs.command_factory: INFO: Built script [/tmp/tmpwwBgVs/job_working_directory/000/2/tool_script.sh] for tool command[cat /tmp/tmpwwBgVs/files/000/dataset_1.dat  > /tmp/tmpwwBgVs/files/000/dataset_2.dat]
galaxy.tools.deps: DEBUG: Building dependency shell command for dependency 'samtools'
galaxy.tools.deps: DEBUG: Find dependency samtools version None
galaxy.tools.deps: WARNING: Failed to resolve dependency on 'samtools', ignoring
galaxy.jobs.runners: DEBUG: (2) command is: mkdir -p working; cd working; /tmp/tmpwwBgVs/job_working_directory/000/2/tool_script.sh; return_code=$?; cd '/tmp/tmpwwBgVs/job_working_directory/000/2'; python "/tmp/tmpwwBgVs/job_working_directory/000/2/set_metadata_gj6rLF.py" "/tmp/tmpwwBgVs/tmp/tmpeSjJLn" "/tmp/tmpwwBgVs/job_working_directory/000/2/working/galaxy.json" "/tmp/tmpwwBgVs/job_working_directory/000/2/metadata_in_HistoryDatasetAssociation_2_xyfvRc,/tmp/tmpwwBgVs/job_working_directory/000/2/metadata_kwds_HistoryDatasetAssociation_2_7VahWW,/tmp/tmpwwBgVs/job_working_directory/000/2/metadata_out_HistoryDatasetAssociation_2_SiN6cX,/tmp/tmpwwBgVs/job_working_directory/000/2/metadata_results_HistoryDatasetAssociation_2_Us0uvr,/tmp/tmpwwBgVs/files/000/dataset_2.dat,/tmp/tmpwwBgVs/job_working_directory/000/2/metadata_override_HistoryDatasetAssociation_2_mYUpFh" 5242880; sh -c "exit $return_code"
galaxy.jobs.runners.local: DEBUG: (2) executing job script: /tmp/tmpwwBgVs/job_working_directory/000/2/galaxy_2.sh
galaxy.jobs: DEBUG: (2) Persisting job destination (destination id: local:///)
requests.packages.urllib3.connectionpool: INFO: Starting new HTTP connection (1): localhost
requests.packages.urllib3.connectionpool: DEBUG: "GET /api/jobs/5729865256bc2525?key=5723299c7bc15b8cb8b00e98a08e9203 HTTP/1.1" 200 None
galaxy.jobs.runners.local: DEBUG: execution finished: /tmp/tmpwwBgVs/job_working_directory/000/2/galaxy_2.sh
galaxy.model.metadata: DEBUG: loading metadata from file for: HistoryDatasetAssociation 2
requests.packages.urllib3.connectionpool: INFO: Starting new HTTP connection (1): localhost
requests.packages.urllib3.connectionpool: DEBUG: "GET /api/jobs/5729865256bc2525?key=5723299c7bc15b8cb8b00e98a08e9203 HTTP/1.1" 200 None
galaxy.jobs: DEBUG: job 2 ended (finish() executed in (619.008 ms))
requests.packages.urllib3.connectionpool: INFO: Starting new HTTP connection (1): localhost
requests.packages.urllib3.connectionpool: DEBUG: "GET /api/jobs/5729865256bc2525?key=5723299c7bc15b8cb8b00e98a08e9203 HTTP/1.1" 200 None
requests.packages.urllib3.connectionpool: INFO: Starting new HTTP connection (1): localhost
requests.packages.urllib3.connectionpool: DEBUG: "GET /api/jobs/5729865256bc2525?full=true&key=5723299c7bc15b8cb8b00e98a08e9203 HTTP/1.1" 200 None
requests.packages.urllib3.connectionpool: INFO: Starting new HTTP connection (1): localhost
requests.packages.urllib3.connectionpool: DEBUG: "GET /api/jobs/5729865256bc2525?key=5723299c7bc15b8cb8b00e98a08e9203 HTTP/1.1" 200 None
requests.packages.urllib3.connectionpool: INFO: Starting new HTTP connection (1): localhost
requests.packages.urllib3.connectionpool: DEBUG: "GET /api/histories/2891970512fa2d5a/contents/5729865256bc2525/display?raw=true&key=5723299c7bc15b8cb8b00e98a08e9203 HTTP/1.1" 200 None
--------------------- >> end captured logging << ---------------------
```
