# Supplementary material for: The Planemo toolkit for developing, deploying, and executing scientific data analyses in Galaxy and beyond
Source: Genome Res. 2023 Feb;33(2):261–8. doi: 10.1101/gr.276963.122 (PMC10069471; doi:10.1101/gr.276963.122)
Supplement: Supplemental Material [file supp_gr.276963.122_Supplemental_Code.tar.gz › planemo-0.75.3/tests/data/tt_success.html]

ID: functional.test\_toolbox.TestForTool\_cat.test\_tool\_000000

Description: Concatenate datasets (for test workflows) ( cat ) > Test-1

Status: success
